# The Effect of Proline on the Freeze-Drying Survival Rate of *Bifidobacterium longum* CCFM 1029 and Its Inherent Mechanism

**DOI:** 10.3390/ijms232113500

**Published:** 2022-11-04

**Authors:** Shumao Cui, Wenrui Zhou, Xin Tang, Qiuxiang Zhang, Bo Yang, Jianxin Zhao, Bingyong Mao, Hao Zhang

**Affiliations:** 1 State Key Laboratory of Food Science and Technology, Jiangnan University, Wuxi 214122, China; 2 School of Food Science and Technology, Jiangnan University, Wuxi 214122, China; 3 National Engineering Research Center for Functional Food, Jiangnan University, Wuxi 214122, China

**Keywords:** proline, *Bifidobacterium longum* CCFM 1029, freeze-drying survival rate, mechanism

## Abstract

Amino acids, which are important compatible solutes, play a significant role in probiotic lyophilization. However, studies on the functions of *Bifidobacterium* during freeze-drying are limited. Therefore, in this study, we compared the freeze-drying survival rate of *Bifidobacterium longum* CCFM 1029 cultivated in different media containing different kinds of compatible solutes. We found that the addition of 21 g/L proline to the culture media substantially improved the freeze-drying survival rate of *B. longum* CCFM 1029 from 18.61 ± 0.42% to 38.74 ± 1.58%. Interestingly, this change has only been observed when the osmotic pressure of the external culture environment is increased. Under these conditions, we found that proline accumulation in this strain increased significantly. This change also helped the strain to maintain its membrane integrity and the activity of some key enzymes during freeze-drying. Overall, these results show that the addition of proline can help the strain resist a tough environment during lyophilization. The findings of this study provide preliminary data for producers of probiotics who wish to achieve higher freeze-drying survival rates during industrial production.

## 1. Introduction

*Bifidobacterium* sp., a common human intestinal probiotic, was first discovered by French pediatrician Tissier in the early 1900s [1]. *Bifidobacterium longum* is an important intestinal probiotic that has been added to many foods and health products to improve atopic dermatitis [2], lactose intolerance [3], and irritable bowel syndrome [4]. Low-temperature vacuum drying is a key technology for producing highly active probiotic powders [5]. However, during the industrial fermentation and production processes, osmotic pressure is the main problem encountered by lactic acid bacteria. The ability to resist changes in the external osmotic environment is of great significance in improving the freeze-drying survival rate [6]. During the whole life cycle of the growth and production of lactic acid bacteria, bacteria are constantly exposed to different environmental pressures. In the face of various survival pressures, such as oxygen, acid, osmosis, and temperature, rapid and effective responses to various pressures are the necessary abilities to survive.

Compatible solutes are polar, soluble, uncharged, small-molecule organics that can accumulate in high concentrations in cells without affecting their function and protein folding under physiological conditions [7]. They mainly include amino acids (glutamic acid, glutamine, proline, etc.) and their derivatives (polypeptides and N-acetylated amino acids), polyols (glycerol), and sugars (trehalose and sucrose). An increase in the osmotic pressure of the environment helps lactic acid bacteria preserve their own structure and maintain the expansion pressure required for cell growth [8]. Tian et al. [9] studied the survival rate of transporter mutants and non-mutants in an environment with increased osmotic pressure, and their results showed that mutants capable of accumulating proline and mannitol were more tolerant to osmotic pressure. Whatmore et al. [10] found that in the absence of any preformed compatible solutes in the medium, *Bacillus subtilis* can adjust its intracellular solute pool by the de novo synthesis of proline to help it adapt to changes in the external culture environment. However, there are few reports on the addition of compatible solutes to the cultivation medium to improve the survival rate of *Bifidobacterium* sp. during freeze-drying, and the underlying mechanisms remain unclear.

In this study, *B. longum* CCFM 1029 was chosen as the model organism and different kinds of compatible solutes were added to the cultivation media to increase the freeze-drying survival rate. The accumulation of the candidate substances was measured using reverse-phase high-performance liquid chromatography (RP-HPLC). We also investigated the underlying mechanisms. The results of this study may provide a new method for improving the freeze-drying survival rate of *Bifidobacterium* sp.

## 2. Results

### 2.1. Effect of Different Substances on the Freeze-Drying Survival Rate of B. longum CCFM 1029

**Figure 1:**
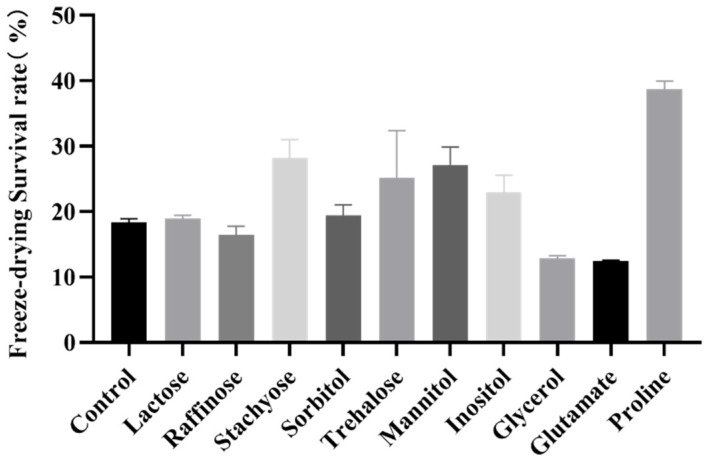
Effects of different substances on the freeze-drying survival rate of *B. longum* CCFM 1029.

The growth medium, an important factor affecting the accumulation of intracellular substances in a strain, may have an important impact on the stability of the strain during freeze-drying. In this study, sugars, alcohols, and amino acids were added to the higher osmotic pressure medium at a concentration of 20 g/L for the fermentation of *B. longum* CCFM 1029. As shown in Figure 1, after adding 20 g/L of proline for fermentation to the stationary phase, the freeze-drying survival rate was significantly improved to 38.70 ± 1.26%, compared with the 18.35 ± 0.45% freeze-drying survival rate of the control group.

### 2.2. Determination of the Optimal Addition of Proline in the Medium

**Figure 2:**
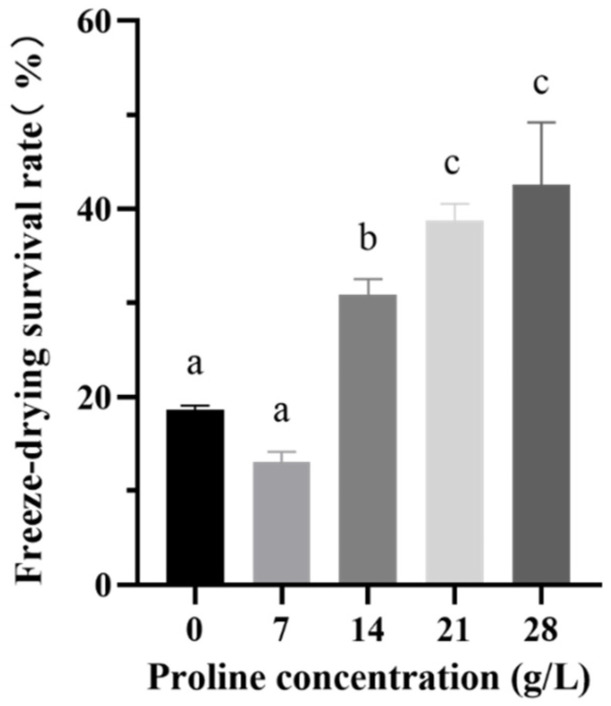
The freeze-drying survival rate of *B. longum* CCFM 1029 after adding different proline concentrations. Columns with different letters indicate significant differences (*p* < 0.05).

The concentrations of proline added to the medium were set to 7, 14, 21, and 28 g/L. As shown in Figure 2, after adding 21 g/L and 28 g/L of proline to the medium, the freeze-drying survival rate of the strain reached 38.74 ± 1.58% and 42.59 ± 5.72%, respectively. These increases were significant (*p* < 0.05) compared with the 18.61 ± 0.42% increase in the control group. However, we selected the 21 g/L dose for subsequent experiments to reduce the cost.

### 2.3. Determination of the Working Conditions of Proline

**Figure 3:**
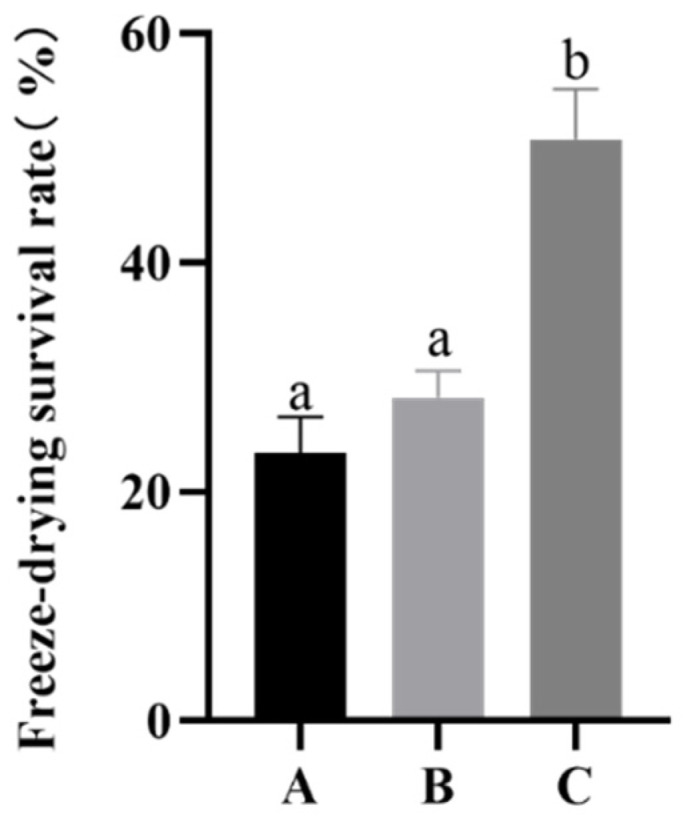
The freeze-drying survival rate of *B. longum* CCFM 1029 under different culture conditions. A is the common osmotic pressure culture group; B is the common osmotic pressure culture group supplemented with 21 g/L proline; C is the higher osmotic pressure culture group supplemented with 21 g/L proline. Columns with different letters indicate significant differences (*p* < 0.05).

Studies have shown that the absorption of compatible solutes by *Bifidobacterium* sp. is an energy-intensive process. Compared with the energy consumption of not absorbing proline, the absorption effect does not seem worthwhile owing to the large amount of energy consumed. We confirmed that proline plays an important role in protecting the strain during freeze-drying. Then, we had to determine which conditions would induce *B. longum* CCFM 1029 to absorb compatible solutes and accumulate them in the cells to improve their freeze-drying survival. As shown in Figure 3, compared to groups A and B, no significant difference was observed in the freeze-drying survival rate of *B. longum* CCFM 1029 with and without proline added under common culture conditions. By comparing groups B and C, it became evident that the freeze-drying survival rate of proline added under higher osmotic pressure culture conditions (50.75 ± 4.42%) was higher than that of proline added under common conditions (28.20 ± 2.35%) (*p* < 0.05).

### 2.4. Contents of Intracellular Proline and Glutamate under Different Culture Conditions

**Figure 4:**
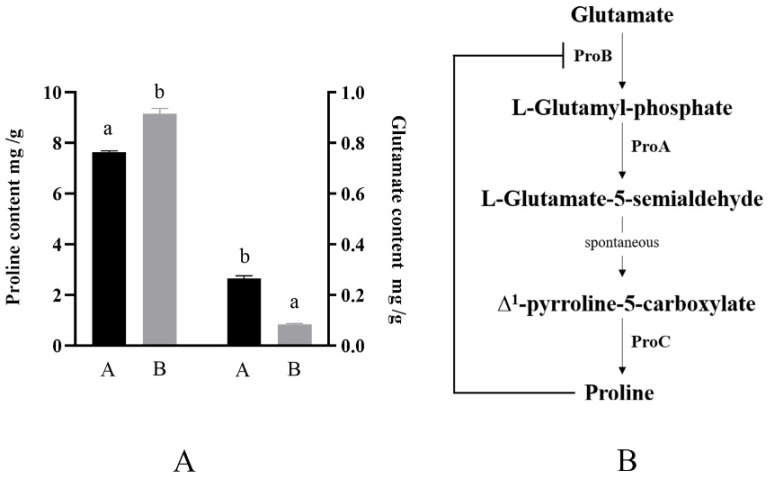
Proline synthesis mode and intracellular proline and glutamic acid contents under different culture conditions. (**A**), *B. longum* CCFM 1029 intracellular glutamate and proline content under different culture conditions. A is the common osmotic pressure culture group with the addition of 21 g/L proline; B is higher osmotic pressure culture group supplemented with 21 g/L proline. Columns with different letters indicate significant differences (*p* < 0.05). (**B**), *B. longum* CCFM 1029 intracellular proline synthesis process.

When the osmotic pressure of the external culture environment increases, the proline accumulation in the cells of the strain is promoted. Therefore, in this study, the proline content in the cells of this strain under different culture conditions was analyzed using RP-HPLC. As shown in Figure 4A, the intracellular proline content of the strain was 9.17 mg/g (wet weight) under higher osmotic pressure culture conditions; this was approximately 1.5 mg/g (wet weight) higher compared with that of the control group. This is consistent with the results of studies on *B. subtilis* [11]. Additionally, we measured the intracellular glutamate content as a synthetic material, which can produce proline (Figure 4B), and found that the glutamate content of the higher osmotic pressure culture group was lower than that of the common osmotic pressure culture group, which further indicated that after the osmotic pressure of the external environment increased, proline was more suitable for accumulation as a compatible solute. Proline accumulated in the cell and helped the strain to resist an unfavorable external environment.

### 2.5. The Candidate Genes Related to Proline Transport and Synthesis

Based on the gene annotation results, the reported genes which may relate to proline transport and synthesis in *B. longum* CCFM 1029 are shown in Table 1. The strain gene sketch was determined, but we found that *B. longum* CCFM 1029 just had *proP*, *putP*, *proA*, *proB*, and *proC*. We identified *proA*, *proB*, and *proC* as candidate genes related to proline synthesis in *B. longum* CCFM 1029, whereas *proP* and *putP* were candidate genes related to proline transport in *B. longum* CCFM 1029.

### 2.6. Expression Changes of Proline Synthesis and Transport Genes

**Figure 5:**
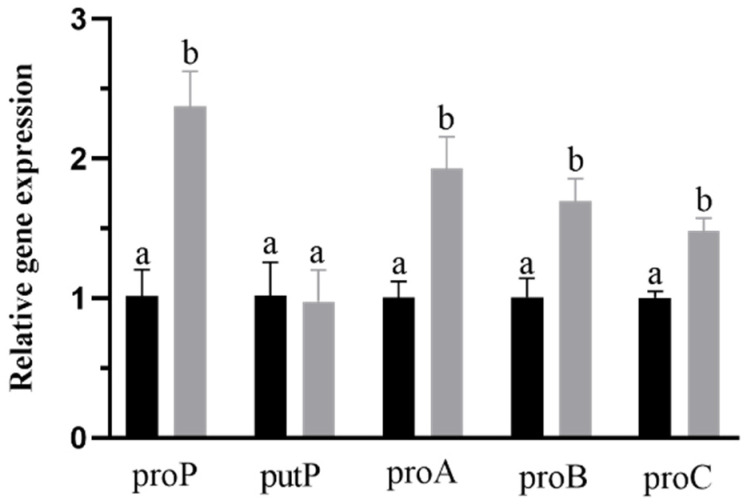
Relative quantitation of genes related to proline synthesis and transport. The black column represents the group of common osmotic pressure cultivation, and the gray column represents the group of higher osmotic pressure cultivation. Columns with different letters indicate significant differences (*p* < 0.05).

After identifying the genes related to proline transport and synthesis in this strain, we used an RT-qPCR to detect the expression levels of related genes in different culture environments. The strain was cultured both in a common osmostic pressure medium and a higher osmostic pressure medium with 21 g/L proline and cultured to the mid-to-late log phase for RNA extraction. The results are shown in Figure 5. Compared with the common osmotic pressure culture group, the expression of *proA*, *proB*, *proC*, and *proP* genes in the higher osmotic pressure culture group increased significantly (*p* < 0.05), indicating that under the condition of higher osmotic pressure, the systems related to proline synthesis and transport in *B. longum* CCFM 1029 were induced. These systems promote the synthesis of intracellular proline and increase the external proline uptake to improve the intracellular proline content of the strain. Interestingly, there was no significant difference in the expression of *putP*, which may be because *putP* is an Na^+^ absolute coupled proline permease, and its expression may be limited by the Na^+^ content in the medium. Compared to *proP*, which is not limited by the environmental Na^+^ content [22], its expression may be restricted by environmental factors.

### 2.7. Effects of Proline on Cell Membrane Integrity

**Figure 6:**
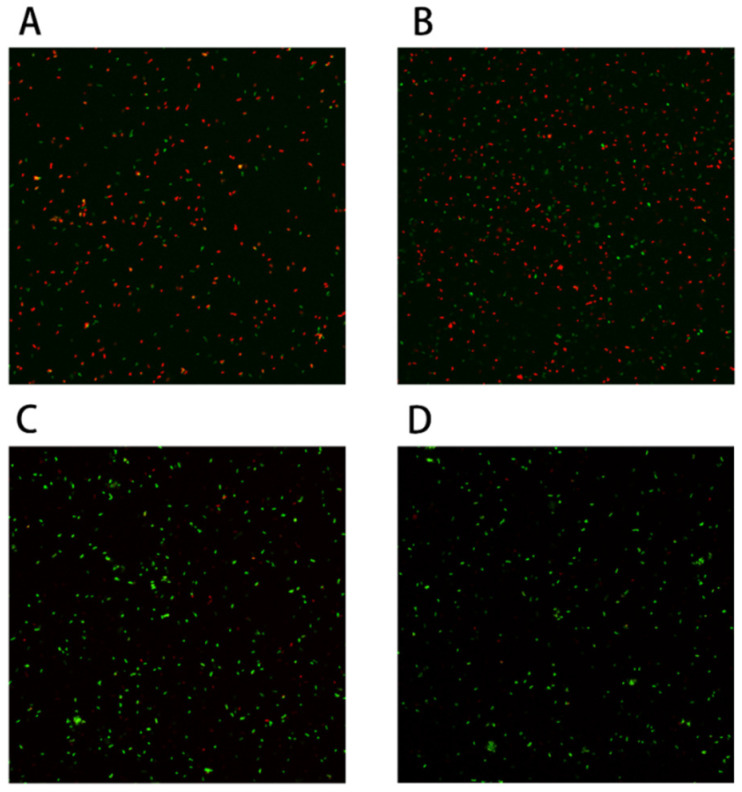
*Bifidobacterium longum* CCFM 1029 cell membrane integrity test: (**A**,**B**) are control groups without proline; (**C**,**D**) are experimental groups with proline.

Many studies have shown that membrane damage is one of the most important causes of strain death during freeze-drying [23]. Therefore, in this experiment we detected the membrane integrity of *B. longum* CCFM 1029 in the experimental group (with proline for fermentation) and control group (without proline for fermentation). If the cell membrane was damaged, this dye would bind to the nucleic acids and would appear red. In contrast, if the cell membrane was integrated, the dye would appear green. Therefore, we could distinguish between strains using this method. As shown in Figure 6, after freeze-drying, the cells harvested by centrifugation from the culture medium containing 21 g/L proline, significantly less red fluorescence was observed under the microscope compared to that in the control group. This indicated that the cell membrane was protected by adding 21 g/L of proline.

### 2.8. Effects of Proline on Intracellular Enzyme Activity

**Figure 7:**
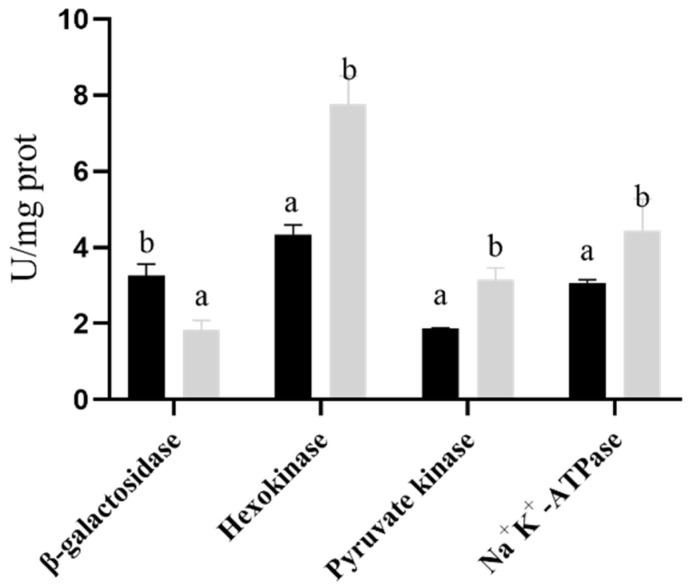
*Bifidobacterium longum* CCFM 1029 intracellular and extracellular enzyme activity test. The black column represents the control group pressure, and the gray column represents the experimental group pressure. Columns with different letters indicate significant differences (*p* < 0.05).

β-galactosidase is an intracellular macromolecular endogenous enzyme that can be used as an indicator of cell membrane changes. The degree of freeze-drying damage to the strain membrane can be evaluated by determining the activity of β-galactosidase in the medium [24]. As shown in Figure 7, the β-galactosidase activity in the control group after freeze-drying was significantly higher than that in the experimental group, indicating that the addition of proline can significantly reduce the cell membrane damage of the strain during the freeze-drying process, thereby increasing the freeze-drying survival rate of the strain. Na^+^-K^+^-ATPase, PK, and HK are the key enzymes involved in the growth and metabolism of the strain. In this study, we evaluated the stability of the strain after proline fermentation by measuring its intracellular enzyme activity. As shown in Figure 7, the higher osmotic pressure fermentation by adding proline significantly reduced the critical effect of enzyme damage on the freeze-drying strain.

## 3. Discussion

In the industry, high cell-density culture is an important way to improve the biomass of lactic acid bacteria. However, in the process of high cell-density production, it is necessary to face the accumulation of higher concentrations of metabolites and by-products. These products will inevitably increase the osmotic pressure of the whole fermentation system, and the growth density of strains is closely related to the osmotic pressure. The continuous increase in the osmotic pressure of the fermentation medium will eventually lead to the stop of bacterial growth. Meanwhile, during the process of vacuum freeze-drying, ice crystals will form with the change of cooling rate and cause mechanical freezing damage to the cell structure. The increase in electrolyte concentration in the unfrozen solution will cause the increase in osmotic pressure. With the continuous water loss of bacterial cells, high osmotic pressure will lead to structural and physiological damage to cells. So, the ability of strains to overcome the increasing osmotic pressure is really helpful for production. However, there is little information about the strategies to improve the ability of *Bifidobacterium* sp. to overcome the increasing osmotic pressure and improve its freeze-drying survival rate.

In this study, the freeze-drying survival rate of *B. longum* CCFM 1029 showed a significant increase after higher osmotic pressure cultivation with the addition of 21 g/L proline. We believe that this may be due to an increase in external osmotic pressure to promote the accumulation of proline. It is well known that an increase in external osmotic pressure causes a large amount of water to flood the bacterial cells instantaneously, disturbing many characteristics of the bacterial cells, and affecting the freeze-drying survival rate of the bacteria [25]. Therefore, during the long evolutionary process, some bacteria have evolved a set of osmotic sensing and regulation mechanisms in response to such external conditions. Cells respond to changes in external osmotic pressure by releasing or accumulating solutes, reducing water flux, and improving the survival rate of the bacteria [26]. It has been pointed out that if the osmotic pressure increases, it regulates the accumulation of solutes through active transport or synthesis [27]. However, this osmotic regulation mechanism has not yet been fully explained, and further research is needed to improve it. We believe that *B. longum* must activate a set of pressure mechanisms under the condition of increased osmotic pressure in the external culture environment, which plays an important role in its freeze-drying process and helps it cope with the harsh environment and improve its freeze-drying survival rate.

We believe that the protective effect of proline on *B. longum* is mainly achieved by protecting its bacterial structure, cell membrane integrity, and key enzyme activities. During the freeze-drying process, the water around the cells as well as inside the bacterial cell membrane is removed; therefore, membrane leakage occurs, preventing the bacterial cells from recovering and restarting growth, which is the main reason for cell inactivation [23]. In this study, the addition of proline protected the integrity of the cell membrane and maintained the cell structure to improve the freeze-drying survival rate.

The addition of proline can also protect the Na^+^K^+^-ATPase, PK, and HK activities of the strain from damage, which may be related to the fact that compatible solutes can protect proteins from protease hydrolysis [28]. Timasheff et al. [29,30] proposed that the binding of compatible solutes to proteins is undesirable and thus is directly excluded from the protein’s hydration shell, which promotes preferential hydration between proteins, which are forced to occupy a smaller volume to minimize their exposed surface; therefore, a more compact, native-like conformation is adopted with increased stability. As the unfolded form would involve more repulsion, making it even more disadvantageous in terms of entropy, the equilibrium shifts toward the folded form, which also increases the stability of the protein. Furthermore, unfavorable interactions between solutes and peptide backbones have been suggested to provide the molecular basis for solute repulsion and subsequent stabilization [31].

Regarding the phenomenon and mechanism of the accumulation of proline as a compatible solute in the cytoplasm when the osmotic pressure of the external environment increased, we used RP-HPLC to clarify its accumulation in cells cultured with higher osmotic pressure. The glutamate content was lower than that of the lower osmotic pressure culture group, while the proline content was higher than that of the lower osmotic pressure culture group. The RT-qPCR was used to determine that the expression levels of genes related to proline synthesis and transport were significantly upregulated. The results indicated that after the external osmotic pressure increased, the bacterial strain increased its intracellular amino acid accumulation by simultaneously increasing its own proline synthesis and external proline uptake and used it to cope with external environmental changes.

## 4. Materials and Methods

### 4.1. Bacterial Strain and Growth Conditions

*Bifidobacterium longum* CCFM 1029 was obtained from the Culture Collection of Food Microbiology, Jiangnan University (Wuxi, China). Based on previous studies (unpublished observations), two special media were used in this study: the common osmotic pressure medium (glucose 20 g/L, yeast extract FM803 17 g/L) and higher osmotic pressure medium (glucose 42.5 g/L, yeast extract FM803 17 g/L). We added 1 mL/L Tween 80, 0.35 g/L MgSO_4_·7H_2_O, 0.05 g/L MnSO_4_·H_2_O, and 1 g/L L-Cysteine to both media. The inoculum of the strain was 5% (*v*/*v*) in order to adjust the initial number of bacteria to reach about 1 × 10^7^ CFU/mL. The medium was cultivated to a stationary phase at a constant pH (5.0) at 37 °C using a triple-tank fermentation control system in an anaerobic (N_2_) environment.

### 4.2. Cell Preparation and Freeze-Drying

The cells were concentrated by centrifugation at 7000× *g* for 20 min, and at 20 °C, and the supernatant was discarded. The cells were washed three times using normal saline (0.85%). The bacterial suspension was prepared by adding a 1:1 (*w*/*v*) protective agent solution (20% trehalose and sucrose (1:1, *w*/*w*)), adjusting the pH to approximately 6.5, and pipetting 1 mL of the solution for freeze-drying.

The parameter setting of the freeze-drying process was shown as the following: control the temperature of the laminate to drop from room temperature to −50 °C within 1 h, and keep it for 4 h (pre-freeze). Control the vacuum degree and adjust the temperature of the laminate to −30 °C within 1.3 h and keep it under 0.2 mbar vacuum degree for 30 h to remove free water (primary drying). Control the temperature of the laminate for 1 h, raise it to 25 °C, and keep it for 20 h under the condition of 20 ubar vacuum (secondary drying).

### 4.3. Determination of the Cell Count of B. longum

The cell counts of *B. longum* CCFM 1029 before and after freeze-drying were performed by the plate dilution method [32] using the MRS-L (plus 0.1% (*w*/*v*) L-cysteine) agar medium at 43 °C and were cultivated at 37 °C in an anaerobic chamber (10% H_2_, 10% CO_2_, and 80% N_2_) for 48 h. When counting viable bacteria after freeze-drying, the cells were suspended in normal saline (0.85%) with the same volume to their original bacterial culture (1 mL). All experiments were performed in triplicate.

$$\mathrm{Freeze}\text{-}\mathrm{drying}\;\mathrm{survival}\;\mathrm{rate}\;\left(\%\right)=\frac{\mathrm{cfu}\;\mathrm{after}\;\mathrm{freeze}\text{-}\mathrm{drying}}{\mathrm{cfu}\;\mathrm{before}\;\mathrm{freeze}\text{-}\mathrm{drying}}\;\times \;100\%$$

### 4.4. Determining the Effect of Different Compatible Solutes on B. longum

In this study, nine substances were selected and added to the fermentation medium, namely, amino acids (glutamic acid and proline), sugars (trehalose, lactose, and stachyose), sugar alcohols (sorbitol, mannitol, and inositol), and glycerin. The medium was prepared according to the above-mentioned formula (the experimental group was supplemented with an additional amount of 20 g/L of compatible solute and the control group was supplemented with glucose to adjust the osmotic pressure to the same to experimental group), and the same amount was placed in biotech-3000 5 L × 3 parallel fermenters. The medium was cultivated to a stationary phase at a constant pH (5.0) and temperature (37 °C) using a triple-tank fermentation control system.

### 4.5. Measuring the Damage to the Cell Membrane

The molecular probe Live & DeadTM Viability/Cytotoxicity Assay kit for Bacteria Cells was used to detect damage to *B. longum* CCFM 1029 before and after freeze-drying. Two groups were both cultivated in the higher osmotic pressure medium.

### 4.6. Measuring the Key Enzyme Activity of B. longum

β-Galactosidase (β-GAL), Na^+^-K^+^-ATPase, pyruvate kinase (PK), and hexokinase (HK) kits were used to measure the enzyme activities of the strain from the control group without proline addition and the experimental group with proline addition. Both of them were cultivated in the higher osmotic pressure medium.

### 4.7. Gene Sketch Sequence Annotation

Glimmer [33]: http://ccb.jhu.edu/software/glimmer/index.shtml (accessed on 9 September 2019), GeneMarkS [34] and Prodigal software were used to predict the coding sequence (CDS) in the genome. tRNAs contained in the genome were predicted using tRNAscan-SE v2.0 [35]. The predicted coding genes were compared with the NR [36], Swiss-Prot [37], and KEGG [38] databases for functional gene annotation.

### 4.8. Design of Primers for Real-Time Quantitative PCR (RT-qPCR)

In this study, the RT-qPCR was used to detect the genes involved in the transport and synthesis of proline in *B. longum* CCFM 1029 after being cultivated in different osmotic pressure culture media. The primer design was based on a genetic sketch of a previously determined strain. The gene and primer sequences were as follows: *proA* (5′-GAGCGATGAATTGAGTCCTGAGGTG; 5′-CCGCAATGGCAAGCAACAGTTC), *proB* (5′-TGCGTTCGGTCGATTCGGTATTC; 5′-TGCGTTCCACATTGCGGTACTG), *proC* (5′-GAGAAGACCACCGCCGAACTTG; 5′-GGCAATCTGGTAGGGCTTGATGG), *proP* (5′-ACGGTCAAATCGCTGCCAAGAG); 5′-AGGTAGGTGAGCACGGTGTAGAAG), *putP* (5′-TGTTCGGCATTCTGGTGGCATAC; 5′-AGTATAGCGAGCACAGCATCAACG). The sequences of the internal reference gene primers EUB338 and EUB518 were synthesized as described by Zhang [39].

### 4.9. RT-qPCR Was Used to Measure the Candidate Genes’ Expression Level

The strain was anaerobically cultivated to the mid-log phase at 37 °C. Bacterial cells were collected by centrifugation and RNA was extracted using a Vazyme Bacteria RNA Extraction kit (Nanjing, China). mRNA was reverse-transcribed into cDNA using a Vazyme SYBR dye kit (Nanjing, China). After preparing all the samples, an RT-qPCR was performed according to the manufacturer’s instructions.

### 4.10. Determination of the Glutamic Acid and Proline Content by UHPLC

Cells were concentrated by centrifugation and then washed three times with normal saline (0.85%). We accurately weighed 1 g of wet cell material and created a 25 mL solution using 5% TCA (*v*/*v*). After filtering, the solution was centrifuged at 12,000× *g* for 30 min at 25 °C, and 400 μL of the supernatant was removed for determination.

The extracted samples were analyzed using an Agilent Hypersil ODS column (5 μm, 4.0 mm × 250 mm) and the ultraviolet detector (VWD) detection wavelength was 338 nm, but proline was detected at 262 nm. Amino acid content was quantified using a 1 nmol/μL amino acid standard.

### 4.11. Statistical Analyses

All experiments were repeated three times. Statistical significance was analyzed by ANOVA and *t*-tests using SPSS (Chicago, IL, USA, V 17.0.0). The statistical significance was set at *p* < 0.05.

## 5. Conclusions

In this study, we analyzed the freeze-drying survival rate of *B. longum* CCFM 1029 after adding different compatible solutes to the cultivation medium. We found that the addition of proline to the medium significantly improved the lyophilization resistance of *B. longum* CCFM 1029 and promoted the intracellular accumulation of proline under higher osmotic pressure culture conditions. When trehalose and sucrose (1:1, *w*/*w*) were used as lyoprotectants, the optimum amount of proline added to the medium was 21 g/L. In addition, when osmotic pressure increased in the environment, the strain accumulated proline both by transporting it from the culture medium and synthesizing it by itself. The addition of proline to the fermentation medium can preserve the cell membrane integrity of *B. longum* CCFM 1029 after freeze-drying and reduce damage to the key enzymes of the strain. This research reveals the inherent mechanism by which proline improves the freeze-drying survival rate of *B. longum* CCFM 1029 after higher osmotic pressure cultivation and provides a reference for the industrial production of probiotic powders.

## Figures and Tables

**Table 1:** The candidate genes related to proline transport and synthesis in *B. longum* CCFM 1029.

Protein	Component	*B. longum* CCFM 1029	Reference
*proU*	*proW*	×	[12,13]
	*proX*	×	
	*ProV*	×	
*YehZYXW*	*YehW*	×	[14,15]
	*YehY*	×	
	*YehZ*	×	
	*YehX*	×	
*OpuA*	*OpuAA*	×	[16,17]
	*OpuAB*	×	
	*OpuAC*	×	
*proY*	*proY*	×	[18]
*putP*	*putP*	√	[19]
*proP*	*proP*	√	[20]
*OpuE*	*OpuE*	×	[16,21]
*proA*	*proA*	√	[11]
*proB*	*proB*	√	[11]
*proC*	*proC*	√	[11]

## Data Availability

All data presented in this study are available in the main body of the manuscript.
